# Evolving technology: creating kidney organoids from stem cells

**DOI:** 10.3934/bioeng.2016.3.305

**Published:** 2016-07-25

**Authors:** Joseph M. Chambers, Robert A. McKee, Bridgette E. Drummond, Rebecca A. Wingert

**Affiliations:** 1 Department of Biological Sciences, Center for Stem Cells and Regenerative Medicine, Center for Zebrafish Research, University of Notre Dame, Notre Dame, IN 46556, USA

**Keywords:** kidney, renal stem cell, organoid, induced pluripotent stem cell, intermediate mesoderm

## Abstract

The kidney is a complex organ whose excretory and regulatory functions are vital for maintaining homeostasis. Previous techniques used to study the kidney, including various animal models and 2D cell culture systems to investigate the mechanisms of renal development and regeneration have many benefits but also possess inherent shortcomings. Some of those limitations can be addressed using the emerging technology of 3D organoids. An organoid is a 3D cluster of differentiated cells that are developed *ex vivo* by addition of various growth factors that result in a miniature organ containing structures present in the tissue of origin. Here, we discuss renal organoids, their development, and how they can be employed to further understand kidney development and disease.

## 1. Introduction

Renal diseases currently affect epidemic numbers of people worldwide, and have continued to escalate in their prevalence globally in recent years, thus representing a significant public health problem for both developing and developed countries. Renal diseases pose immediate, life-threatening medical consequences because the kidneys are needed to carry out a battery of essential tasks that cooperatively maintain the daily homeostasis of the body. Humans have a pair of bean-shaped kidney organs that are responsible for vital functions that include (i) the excretion of metabolic wastes and other toxins, most notably being urea, (ii) the regulation of body fluids, which entails the precise control of osmolarity, acid-base balance, and electrolyte levels, and (iii) the production of various hormones and enzymes that regulate such processes as the control of blood pressure and erythrocyte maturation within the bone marrow [1]. As such, kidneys are incredibly complex organs, with their diverse functions reliant on the proper development and maintenance of over twenty different epithelial and mesenchymal cell types [2].

### 1.1. Stages of the developing mammalian kidney

**Figure 1:**
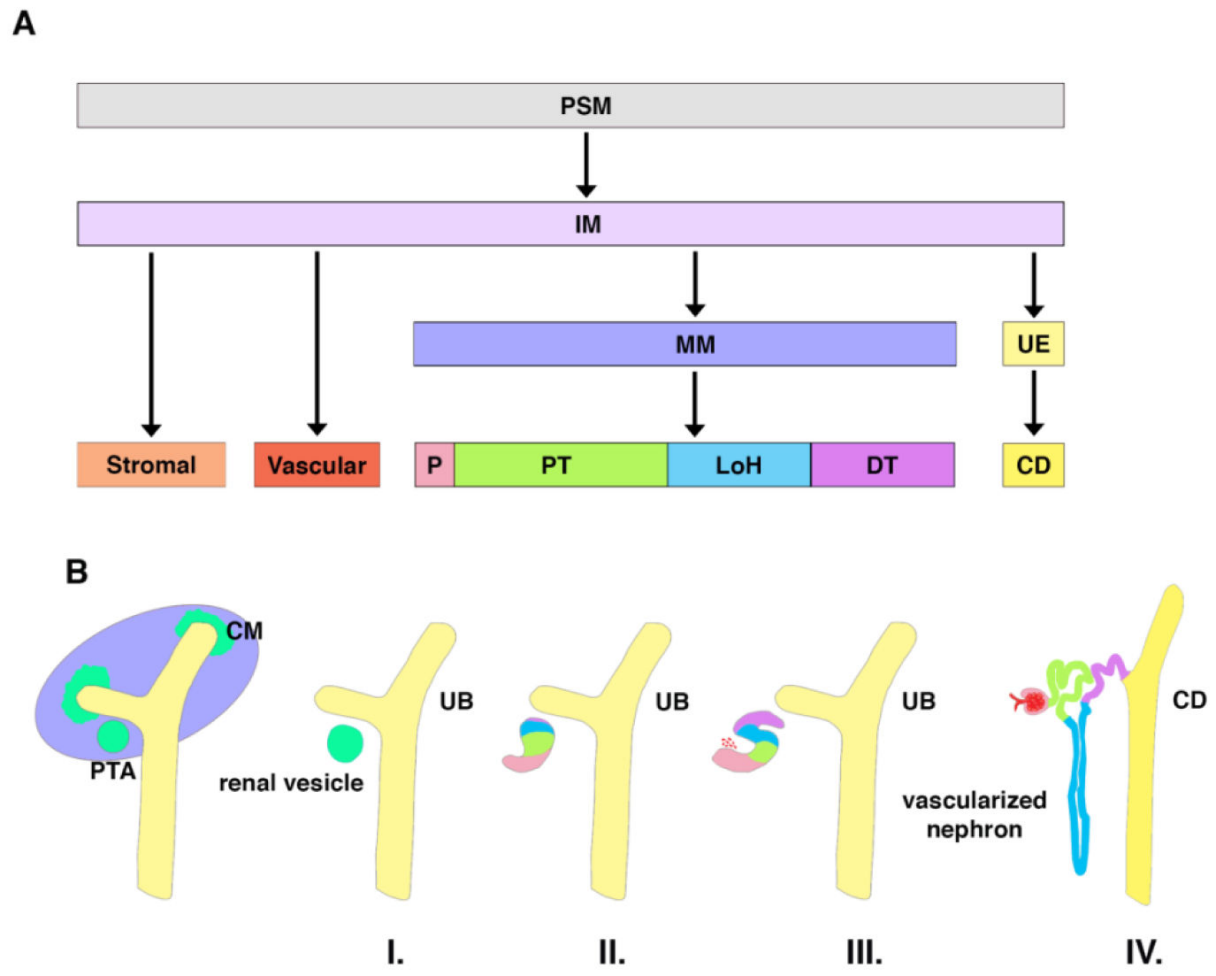
The lineages and anatomical stages of the developing mammalian kidney. A) Cell lineages are depicted beginning with the presomatic mesoderm (PSM) which gives rise to the intermediate mesoderm (IM). The IM will differentiate into stromal, vascular, metanephric mesenchyme (MM), and ureteric epithelium (UE). MM develops into the podocyte (P), proximal tubule (PT), Loop of Henle (LOH), and distal tubule (DT), while the UE results in the collecting duct (CD). B) Anatomical stages are shown first with condensation resulting in cap mesenchyme (CM) and pretubular aggregates (PTA) around the ureteric bud (UB). CM and PTA are followed by formation of the renal vesicle (I), comma-shaped body (II), S-shaped body (III), and vascularized nephron (IV).

During embryogenesis there are three major germ layers: the endoderm, mesoderm, and ectoderm. Together these layers give rise to the initial tissues and structures that are further refined during development to create an organism. The mammalian kidney develops from the mesoderm, specifically presomatic mesoderm (PSM), also termed primitive streak, in a spatiotemporal manner (Figure 1) [3,4]. Cells from the PSM rely on Wnt signaling to migrate rostrally, forming the intermediate mesoderm (IM). At this point, retinoic acid (RA) signaling is imperative for anterior-posterior specification of the IM. Anterior IM forms the ureteric epithelium (UE), then later the ureteric bud (UB), which subsequently forms the collecting duct (CD). Additionally, posterior IM gives rise to metanephric mesenchyme (MM) after longer exposure to Wnt signaling [3].

As development progresses, the MM responds to elaborate signals from the UB that induce its condensation and mesenchymal-to-epithelial transition (MET) [5]. Progressive reciprocal crosstalk of these components leads to the elaboration of a branched kidney structure. During early nephrogenesis, the condensation of MM forms cap mesenchyme (CM) (Figure 1). Subsequently, the CM will be induced to form pre-tubular aggregates (PTAs). From each PTA, a renal vesicle will form (Stage I), which develops into first a comma-shaped body (Stage II), then an S-shaped body (Stage III), and eventually the segmented nephron (Stage IV) [6,7]. Vascular progenitors, stromal progenitors, and CM are three MM cell types that later form the nephron and its supporting microenvironment [7]. The nephron is the functional unit of the kidney that is composed of a glomerulus, epithelial tubules, and collecting duct [8]. The nephron maintains homeostasis by filtering blood, reabsorbing water and nutrients, while secreting waste in the form of urine [9].

### 1.2. Models used to study the kidney: 2D cell culture and animal systems

Biomedical research has largely employed 2D cell culture and animal models to interrogate development and perform drug testing. Currently, each model has limitations with regard to determining the toxicity and efficacy of a drug before it reaches clinical trials [10]. Partly as a result of this, the cost of developing new pharmaceuticals is enormous [11,12]. A study by DiMasi and colleagues in 2003 calculated a drug’s average time in clinical trials to be 9 years with an average cost of over 800 million dollars [11]. More recently, DiMasi et al. published an updated version of the study for drug development indicating a drastic increase in price of taking a drug from discovery to the clinic [12]. They calculated that drug development now costs approximately 2.56 billion dollars in pre-approval costs [12].

In the nephrology field, there remains a tremendous need in particular to understand more about renal toxins, as they cause 30–50% of acute kidney injury cases [13,14]. However, while cell culture is fairly simple and affordable, it fails to replicate the complexities of an *in vivo* system, and usually needs extremely high concentrations to result in any toxic effect [15–17]. Although animal models can address some of the limitations inherent with 2D culture systems, they are more expensive, pose ethical issues, and do not always replicate what is seen in humans. While human patients are from varied genetic backgrounds, have different environmental factors, and are different ages, many studies employing animal models do not have ways to address these complex levels of diversity [10].

Specific examples of these models and their shortcomings can be illustrated. A study completed by Jenkinson and colleagues (2012) highlights some weaknesses of using cell lines to test toxicity in the nephron [18]. Several shortcomings of the HK-2 proximal tubule cell line are illustrated including inconsistent expression of known transporter genes [18]. Furthermore, another study used HK-2 cells and primary human renal proximal tubule epithelial cells to compare expression levels of three accepted biomarkers of nephron damage (KIM-1, NGAL, M-CSF). This study found discrepancies between the cell lines and between the biomarker signatures as well [19]. Greek and Menache (2013) noted several weaknesses in the translatability of animal models [20]. For example, HIV and neuroprotective drugs have been repeatedly discovered to work in animals, while their benefits were not translated to humans [20]. Taken together, the need for other nephrotoxicity testing methods is evident. One such method has come to light in recent years. This new technology of generating an organoid has endless possibilities from nephrotoxicity assays, developmental investigations, transplantation possibilities, and many more.

**Figure 2:**
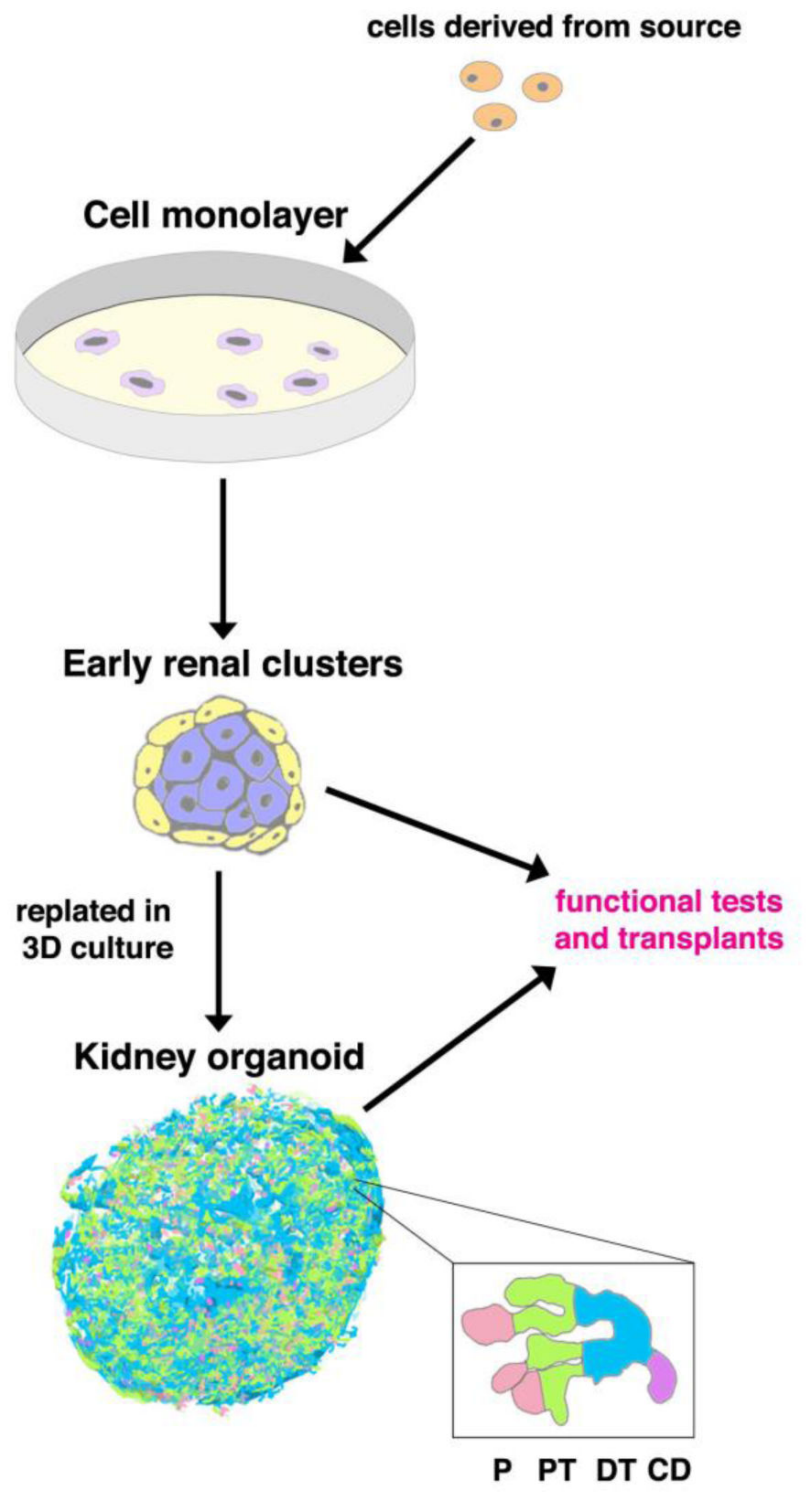
General schematic of an organoid creation procedure. Cells are harvested from the cell source, such as induced pluripotent stem (iPS) cells. These cells are plated in monolayer cell culture. Subsequently, they form early renal clusters when they are replated into 3D culture with the appropriate media conditions, where they spontaneously form organoids, depicted here as a renal organoid complete with podocyte (P), proximal tubule (PT), distal tubule (DT), and collecting duct (CD) segmentation. There are typically functional tests and transplants performed to determine efficacy of the protocol.

Organoids can be described as a 3D cluster of cells that resemble a particular organ both genetically and functionally. An organoid is constructed by first isolating cells from a source, plating these cells in 2D culture, adding combinations of growth factors to mimic *in vivo* development, and replating the cells in 3D culture where spontaneous organoid formation is observed (Figure 2). After organoid formation, testing functionality is crucial to validate the resulting product’s physiological behavior. Many advances have been made in various organ systems, however the complexity of the kidney has delayed advancement in this specific field of research. Here, we discuss how researchers have generated organoids that mimic *in vivo* nephrogenesis.

## 2. Creation of Kidney Organoids Through Re-Aggregation Approaches

A major breakthrough that inspired later research on organoids was the discovery that certain tissues and organs could be dissociated through enzymatic digestion to permit single cell culturing. More importantly, when reaggregated, these cells would form structures similar to those seen in their complex tissue of origin [21–28]. In 2010, Unbekandt and Davies demonstrated that embryonic kidneys harvested from mice could be dissociated and reaggregated via centrifugation to form renal structures such as immature glomeruli and tubule rudiments [29]. Mouse kidneys at stage E11.5 that had not undergone nephrogenesis in the developing embryo were used as a cell source. Immediately after reaggregation, both mesenchymal and epithelial cells were identified based on calbindin and E-cadherin double positive and double negative staining. Yet, more advanced structures were absent. After being cultured for three days in medium supplemented with the ROCK inhibitor H1152, a large degree of calbindin positive epithelial cells formed branched structures and produced a laminin positive basement membrane, indicative of UB epithelia. Interestingly, removal of the ROCK inhibitor after 24 hours in culture led to the formation nephrogenic structures such as comma-shaped and S-shaped bodies, identifiable by their characteristic morphology. Additional mature structures were also identified based on the expression of Megalin for proximal tubules, E-cadherin for distal tubules, and *Wt1* expression at the glomerular pole. The authors proceeded to knockdown *Wt1* with siRNA and showed that re-aggregated cells formed fewer nephron structures, but produced UB epithelia in the same manner as untreated cells, mirroring previous studies showing that genetic knockouts of *Wt1* causes renal agenesis [29–31].

In order for renal organoids to be of use in clinical settings, it is important that they be able to form essential nephron functions. Further, the ability for these organoids to integrate with the host vasculature in a safe and efficient way would likely be necessary. Xinaris and colleagues undertook a study to test the ability of organoids derived from mouse embryonic kidneys to form functional organoids *in vivo* after transplantation into rats [32]. First, E11.5 embryonic mouse kidneys were enzymatically dissociated and single cells were cultured and pelleted. These cells produced UB epithelium expressing calbindin and a laminin positive basement membrane. Nephrogenic structures were also observed including renal vesicles, comma-shaped bodies, and S-shaped bodies based on morphology and the presence of Ncam and Pax2 proteins. While *Wt1^+^* cells were present at the glomerular pole of nephron structures, mature glomeruli with properly developed slit diaphragms were not observed. The researchers achieved implantation of the cellular suspensions by growing cells *in vitro* for five days, then transplanting the clusters underneath the renal capsule of athymic rats. The organoids were allowed to grow in this setting for three weeks and subsequently showed many tubular structures and histological evidence for immature glomeruli. To increase the efficiency of glomerular differentiation, organoids were supplemented with VEGF after transplantation into rats. VEGF was found to increase the degree of vasculature within the organoids based on RECA-1 and α-SMA immunohistochemistry. More importantly, the robust formation of mature glomeruli was noted based on expression of the slit diaphragm marker, claudin-1 and vascular positive structures within Wt1^+^/Synaptopodin^+^/Nephrin^+^ glomeruli. Also, the functionality of the glomeruli was shown to be intact as these structures contained red blood cells, and they were able to take up fluorescently conjugated BSA and dextrans injected into the host vascular system [32]. However, the need for continuous treatment of organoids with chemicals in order to obtain the proper formation of physiological structures is a limitation of this method for clinical applications [32].

In a follow up study, Xinaris et al. considered the ability of amniotic fluid stem cells (AFSCs) to induce the formation of mature glomeruli within murine embryonic kidney derived organoids [33]. First, more in-depth analyses were performed to characterize the sub-structure of glomerular components within the organoids formed using their previous method of initial differentiation in culture followed by implantation under the renal capsule of athymic rats. Electron microscopy showed the presence of immature podocytes as well as more mature structures containing interdigitating foot processes and filtration slits. Intricate assessment of function was performed using injection of fluorescent conjugated dextrans of large (155 kDA), small (10 kDA), and intermediate sizes (70 kDA) into the host vasculature. Under normal physiological circumstances the glomerulus is able to filter dextrans and other molecules up to 70 kDA in size [34]. While all of the dextrans were able to reach the glomeruli within the transplanted organoids, indicating proper connections with the host vasculature, only 10 kDA and 70 kDA conjugated dextrans were observed within the proximal tubules. Furthermore, the dextrans that entered into the tubular compartments were found to co-label with Megalin. After performing these analyses, the authors utilized transgenically modified human AFSCs expressing glial cell line-derived neurotrophic factor (GNDF), known for its role as a key regulator of the MM. After two days of integrated culture, AFSCs were found in PAX2^+^ structures within the organoids. AFSCs were present in glomerular structures, specifically a podocyte lineage, and capable of establishing foot processes based on expression of α-actin-4, Podocin, and human specific Nestin [33].

These chimeric organoids hold great promise for future studies of diseases, cell autonomous processes, and clinical uses because they do not need continuous treatment with exogenous chemical factors. However, questions remain based upon the method and animal model used in these studies. One of these questions involves the use of embryonic serum in media, which contains a number of growth factors, whose exact role in the differentiation and survival of organoids remains unknown. These questions can also be applied to signals, which may originate from the host environment, but as of yet remain unidentified. Secondly, these studies were performed in athymic rats, which fail to produce T-cells and therefore do not have adverse reactions to cells from other exogenous sources. The use of induced pluripotent stem (iPS) cells to produce renal organoids would provide further benefit and circumvent the need for anti-rejection medications in clinical settings.

## 3. Creation of Kidney Organoids Using Different Sources of Stem Cells

As previously described, specific renal lineages arise during ontogeny when the IM differentiates into MM, from which nephron cell types emerge, or into the UB lineage which forms the collecting duct system. Xia et al. reported a method to create UB *in vitro* [35]. This project began with iPS cells that were reprogrammed from human fibroblasts. Then, they established a four day protocol using both iPS and embryonic stem (ES) cells in monolayer while adding several growth factors including BMP4 and FGF2 to lead the cells to a mesoderm-committed fate. They would then add BMP2, RA, and Activin A that resulted in IM and renal lineages. The mRNA expression data from these cells showed the closest match to UB-like cells. Next, Xia et al. used reaggregation assays with murine embryonic kidneys to find mouse MM cells were sufficient to enable iPS to UB-like cells. The researchers were further able to direct patient derived polycystic kidney disease iPS cells down a renal lineage and form chimeric UB structures. This research played a critical role in advancing the field of renal organoid studies [35].

Lam et al. (2013) developed a technique to efficiently differentiate human ES and human iPS cells into cells that express IM and renal tubular structure markers [36]. After using immunofluorescence and quantitative PCR, Lam et al. found that a Wnt agonist, CHIR works best to mimic gene expression during gastrulation leading to proper development through primitive streak with consistent epithelial-to-mesenchymal transitions (EMT). After adjusting CHIR treatment, the scientists conclude that CHIR duration is important as it results in varied endoderm levels. From there the authors screened growth factors that would result in IM and found that FGF2 is able to induce *PAX2* expression in CHIR treated cells. Combining FGF2 with RA results in *PAX2^+^LHX1^+^* cells, both markers of IM. After optimizing the protocol with these added factors, they tested the procedure on three human ES cell lines and two human iPS cell lines where they found all but one iPS line had over 80% of *PAX2^+^LHX1^+^* cells. RT-PCR results showed expression profiles of these cells were consistent with data of high IM gene expression on day three, which then decreased on day five. Inversely, WT1 expression increased from day three to day five. Similarly, both *PAX2^+^LHX1^+^* iPS cell lines and ES cell lines show decreased expression of IM markers, while epithelial tubules form that stained positive for LTL, KSP, and N-cadherin. Other markers including those of the podocytes, collecting duct, proximal tubules, and loop of Henle were identified by immunostaining.

To further developing their protocol to increase CM levels, Lam et al. screened a number of growth factors as well [36]. Using immunostaining they found that FGF9 and Activin A increased *SIX2* expression, a marker of CM. Staining for two other CM markers, *SALL1* and *WT1*, further supported this increase in CM. During kidney development, CM induction is caused by Wnt signaling. Lam et al. mimicked this phenomenon using *SIX2*^+^ cells and adding CHIR on day six of differentiation. The CHIR treatment resulted in decreased *SIX2* expression and increased LTL expression indicating mature cells. While Lam and colleagues did complete a reaggregation study, similar to previously mentioned studies, they did not test the functionality of their products [36]. Testing for kidney-like function would add another layer of complexity and support the theory that the identity of these structures is truly kidney [36].

In a follow up study, Morizane et al. (2015) describes their system for differentiating human iPS and ES cells into what they term ―nephron progenitor cells‖ (NPCs) via mimicking metanephric development [37]. These NPCs are multipotent and can further differentiate into several nephron tissue types. Throughout their article they detail how they optimized the concentrations and durations of chemical treatments. Beginning with the stem cells, ES cells were treated with 8 uM CHIR, while iPS cells were augmented with 10uM CHIR and 5 ng/ml noggin treatment. This treatment resulted in *T^+^TBX6*^+^ late primitive streak cells that were treated with Activin from day four to day seven developing into *WT1^+^HODX11^+^PAX2^−^LHX1*^−^ cells, indicative of IM.

Next, the researchers wanted to create MM and found the addition of FGF9 results in *SIX2*^+^ cells [37]. They used immunocytochemistry and flow cytometry to determine that other MM markers, SALL1, WT1, and EYA1 present in these cells. Quantitative PCR revealed *OSR1* levels remained constant in these cells from days 7–9 differentiation. However, SIX2 expression decreased between days 10–14 as these NPCs differentiated into clusters of *PAX8^+^LHX1*^+^ cells, defined by the authors as renal vesicles. At this point Morizane et al. kept some cells in 2D culture while also replating some NPCs in 3D round bottom wells and removed FGF9. Both 2D and 3D cells formed clusters that were *LTL^+^NPHS1^+^PODXL*^+^. The removal of FGF9 shows these cells were intrinsically programmed to differentiate into nephron structures. Initially this process was not efficient, but upon further optimizing the procedure they were able to produce 76% *PAX8^+^LHX1*^+^ cells that organized into clusters. With no additional factors added, by day 21 of differentiation the clusters formed nephron structures that stained positive for markers of distinct cell types including glomerulus, proximal tubules, distal tubules, and Loop of Henle, with collecting duct noticeably absent. The absence of collecting duct shows this method is specific for NPCs, the result of MM. However, the study does not illustrate the ability to form a complete kidney with all cell types.

Interestingly, all organoids formed lasted for a minimum of 56 days and they were over 20 times more efficiently constructed compared to the lab’s previous protocol [36]. These organoids were also tested for function to further prove the inherent differentiation of the cells. Morizane et al. used DAPT to inhibit notch signaling, resulting in a dysfunctional system. The organoids were treated with gentamicin or cisplatin, known nephrotoxins, and assayed for functionality. The gentamicin injured proximal tubules in a dose dependent manner as determined by real time PCR levels of kidney injury molecule-1 (*KIM-1*). Using γH2AX, a DNA damage marker, the researchers found cisplatin treatment also damaged proximal and distal tubules in a dose dependent manner [37].

In a similar study completed by Takasato et al. (2014) human ES cells (hESCs) were used to generate kidney organoids [38]. In order to induce the formation of primitive streak from hESCs, the cells were treated by one of two methods. In the first method, hESCs were treated with BMP4 and Activin A, which are key signaling molecules known to be important for the formation of the primitive streak *in vivo*. The second technique relied upon stimulation of canonical Wnt signaling with the chemical CHIR. Both approaches resulted in robust formation of primitive streak based upon expression of MIXL1, T, and SOX17 and led to spontaneous differentiation of the primate streak tissue into IM. However, while robust expression of *OSR1* was noted, other markers such as *PAX2* and *LHX1* were absent based on PCR and immunofluorescence, indicating the need for further refinement of the differentiation method.

Based on its physiological role, posterior streak induced hESCs were subsequently treated with fibroblast growth factors (FGFs) [38]. FGF2 or FGF8 caused the cells to give rise to IM expressing *PAX2* and *LHX1* after four days of culture. A direct role for FGF signaling was shown by knockdown of FGFR1 and FGFR3 that inhibited IM induction. To further direct differentiation potential to a nephrogenic fate, IM was treated with FGF9, BMP7, and RA for an additional 12 days. This allowed for differentiation into MM, which was identified based on *SIX2, WT1, GDNF*, and *HOXD11* indicating the presence of tubular progenitors, renal stroma, and nephric duct. At day 14 *ECAD^+^PAX2*^+^ epithelial structures were surrounded by *SIX2^+^WT1^+^* mesenchyme [38]. This represents a mesenchymal field that contributes to nephrogenesis in the developing kidneys of the embryo [5]. At day 22, the expression of mature nephron makers was present based on RT-PCR including makers for podocytes (*SNPO, NPHS1* and *WT1*), proximal tubules (*AQP1* and *SLC3A1*), as well as collecting ducts (*AQP2* and *SCNNB1*). At this point, RA and FGF treated posterior streak generated from treatment with CHIR created robust UE at the expense of MM.

In order to determine the ability of single cells to reproduce nephrogenic structures, mouse embryonic kidneys were dissociated and the cell suspensions mixed with hESCs that had undergone the differentiation protocol to day 12 [38]. Upon sectioning of aggregates cultured for four days, the hESCs produced via CHIR and FGF9 were shown to integrate into all major structures of the developing kidney including UE (*PAX2*^+^*CALB^+^*), renal vesicle (*CDH6^+^JAG1*^+^) and progenitor mesenchyme (*SIX2^+^WT1^+^*). However, hESCs that had undergone differentiation with the sequential treatment of BMP4 and Activin A, followed by FGF9 treatment, and finally with exposure to FGF9, BMP7, and RA only contributed to UE and MM. Taken together, these results illustrate the ability of hESCs to successfully differentiate and form nephrogenic and mature nephron structures [38]. An advantage of this technique is that mutations can be introduced at distinct stages of development and the resulting effects can be observed. Unfortunately, this study did not investigate the maturity of glomerular structures or functionality of tubular components, and vasculature was not noted [38].

Takasato et al. (2015) adapted their technique from the previously described study (2014) to accomplish kidney differentiation from human iPS and ES cells [3,38]. Their adaptations were focused on further understanding the mechanisms regulating induction of collecting duct compared to kidney mesenchyme progenitors. The end result was a renal organoid complete with collecting duct, renal interstitium including indications of vasculature, and epithelial cells. The authors use quantitative PCR to illustrate that kidney mesenchyme progenitors are the result of MM, derived from posterior IM, the fate of presomitic mesoderm cells when exposed to Wnt signaling longer. Accordingly, the presomitic cells exposed to Wnt via CHIR for a shorter duration resulted in anterior IM, followed by UE, resulting in collecting duct.

Using this information, Takasato et al. altered their procedure to increase the MM formed in both iPS and ES cells [3]. After four days of CHIR treatment, FGF9 was added to the MM and UE in monolayer and the mixtures were transferred to organoid culture where they were cultured for 20 days and formed kidney organoids. Immunofluorescence showed organized clusters of kidney cell types including collecting duct, early distal tubule, early proximal tubule, and glomerulus. Using confocal microscopy and *z*-stack images, the authors illustrate correct 3D positions of the developing nephrons with collecting duct at the bottom and glomerulus at the top. Furthermore, RNA sequencing on whole organoids was performed at days 0, 3, 11, and 18. While observing the expected inverse relationship of renal progenitors and segment markers, the authors also noted that organoids from days 11 and 18 were comparable to the RNA transcript profile of a first trimester human embryonic kidney [3].

Next, Takasato et al. showed that the developing kidney organoids displayed unique characteristics [3]. Some of these included immunofluorescence of developing proximal tubules, glomeruli with podocytes, and renal interstitium. Further detail is shown with TEM, such as to show tight junctions in distal tubules, brush borders in proximal tubules, and foot processes in podocytes. After showing presence of these fine details, the researchers performed functional tests of the organoids. Taking advantage of the prime reabsorption ability of the proximal tubules, Dextran-Alexa488 was endocytosed by the tubules assumed to be proximal tubules (LTL^+^) exhibiting the functionality of these organoids. Another example of a conserved physiological response was testing the apoptotic result of cisplatin treatment. As expected, the mature proximal cells underwent apoptosis after being treated with cisplatin. Taken together, these two tests illustrate that these organoids are a potential tool for future uses such as renal toxicity assays, modeling kidney development and diseases, and cell therapies [3].

**Figure 3:**
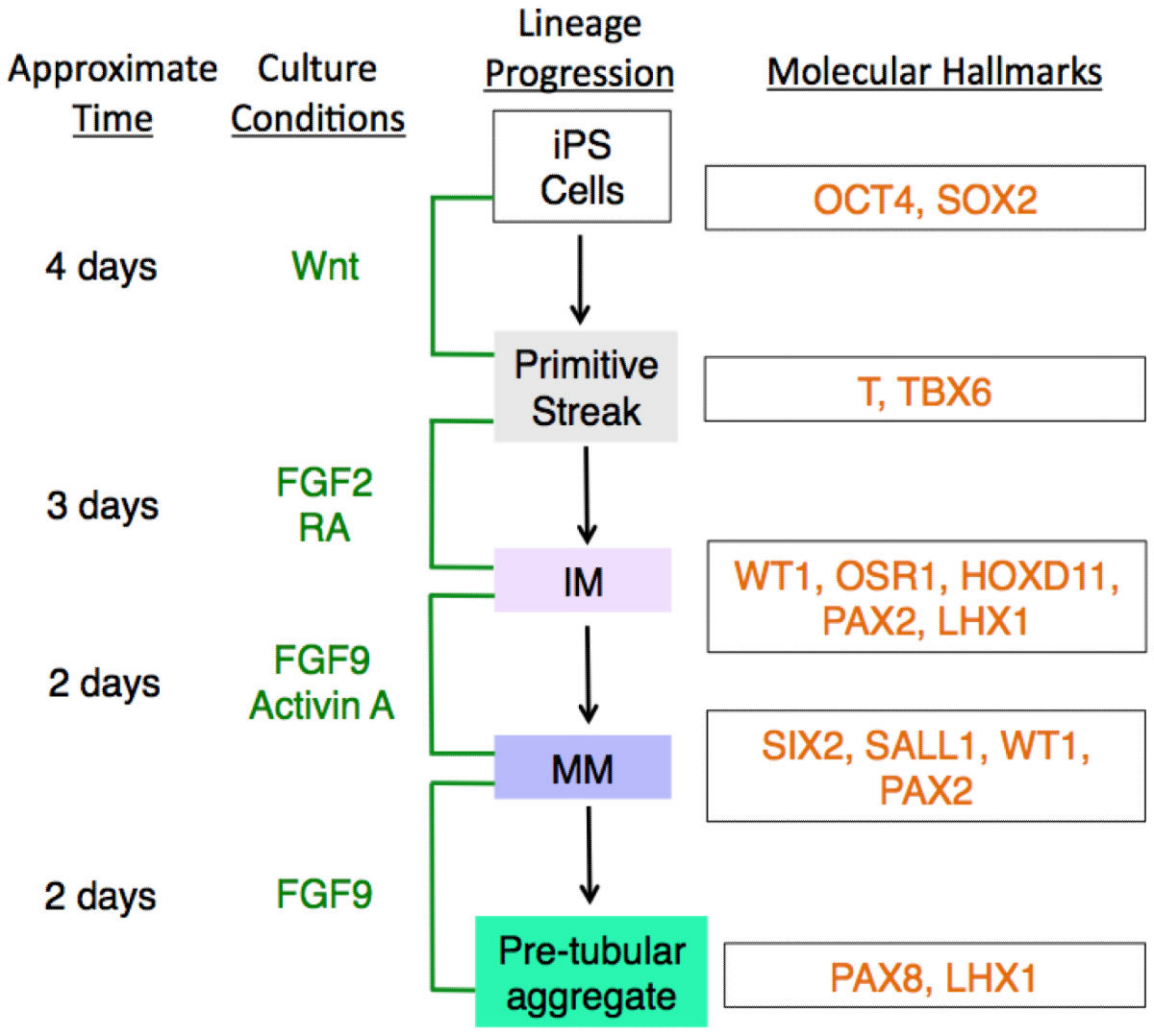
A working model of current common intermediate differentiation steps used to guide pluripotent stem cells to adopt a renal lineage *in vitro* for creation of kidney organoids. The schematic outlines the approximate time interval for each progressive culture condition and the resulting lineage specification of the cells that is associated with the sequence of provided factors, where the lineage state is surmised based on the accrual of particular combinations of molecular hallmarks. Beginning with OCT4^+^SOX2^+^ iPS cells, modulation of Wnt signaling for four days induces the cells to adopt a primitive streak identity based on expression of T and TBX6. Upon subsequent FGF2 and RA treatment for three days, intermediate mesoderm (IM) cells result, which express WT1, OSR1, HOXD11, PAX2, and LHX1. After this, two days of treatment with FGF9 and Activin A leads IM to metanephric mesenchyme (MM) that is distinguished based on expression of SIX2, SALL1, WT1, and PAX2. Following the removal of Activin A but continued exposure to FGF9 for 2 additional days, the MM is observed to form pre-tubular aggregates that express PAX8 and LHX1. This model is largely based on schemes reported by references [3,36–38].

In summary, the findings reported by several independent research groups to date have indicated very similar steps in the directed formation of renal organoid structures from iPS cells, thus cumulatively suggesting a shared working model for this procedure (Figure 3) [3,36–38]. This common workflow entails the progressive exposure of the cell source using a combination of different growth factors in particular sequences and for defined durations to lead to comparable outcomes to coax them to renal lineages. The critical series of culture conditions involves factors that modulate Wnt signaling, followed by combinations of FGFs and RA signaling (Figure 3).

## 4. Conclusion

The complexity of the kidney offers tremendous difficulty to the field trying to study this vital integral organ. With advances in technology such as organoids, researchers have a new opportunity to explore critical questions that may not be appropriately addressed or answered with currently existing models. As the refinement of these organoids evolves, the number of possible applications grows. The easily accessible nature of organoids positions them for use in therapeutic studies.

One major application of kidney organoids will be their use for nephrotoxicity assays. Due to the similarities between 3D kidney cell clusters and *in vivo* nephrotoxic results, many believe organoids can provide a better model than using 2D cell culture and animal models [39,40]. Another benefit of using organoids compared to 2D cell culture is the ability to test chronic exposures of potential nephrotoxins [10]. Kidney organoids have also been combined with nanoparticles in a nephrotoxicity assessment, something that would not be as complete of a study using a 2D cell culture system [41].

Disease modeling with organoids has shown promise as well. Batchelder et al. (2015) were able to sustain 3D organoids of human renal cell carcinoma in culture for 21 days. This enables a new avenue for testing patient specific cancer treatments [42]. Also, using intestinal stem cell organoids in combination with the CRISPR/CAS9 system, scientists were able to correct the cystic fibrosis transmembrane conductor receptor (CFTR) [43]. Similarly, the CRISPR/Cas9 technology has been used to induce mutations in organoids to study developmental disorders using kidney organoids [44]. Combining organoid technology with other innovative techniques like CRISPR/CAS9 and nanoparticles is only the beginning of the possible studies that can help further understand the complexity of kidney development and disease.

One important goal in developing kidney organoids is the prospect that this technology could be used for personalized medicine. For example, a patient’s own cells could be converted to iPS cells, and then differentiated down a lineage with the intention of kidney replacement or individualized nephrotoxicity assays [45]. This would avoid long wait times on donor lists, the need for life-long dialysis treatments, and potentially reduce the risk of acute kidney injury. Further refinement will be needed before the field reaches the point of human kidney organoid transplants. One such area for further research should focus on biomechanical stresses that the transplanted organoid may encounter *in vivo*. For example, how will this transplanted organoid respond to blood pressure? Does the anatomical position in the human body affect signaling within the kidney? While these questions and many more will need to be answered before kidney organoids could be applied in clinical settings, there is no doubt that organoid technology has endless possibilities. These possibilities are sure to occupy the field of kidney research for the near future.
